# Proteome data of *Anopheles stephensi* salivary glands using high-resolution mass spectrometry analysis

**DOI:** 10.1016/j.dib.2018.11.070

**Published:** 2018-11-27

**Authors:** Gourav Dey, Ajeet Kumar Mohanty, Sreelakshmi K. Sreenivasamurthy, Manish Kumar, T.S. Keshava Prasad, Ashwani Kumar

**Affiliations:** a Center for Systems Biology and Molecular Medicine, Yenepoya Research Centre, Yenepoya (Deemed to be University), Mangalore 575018, India; b Institute of Bioinformatics, International Tech Park, Bangalore 560066, India; c Manipal Academy of Higher Education, Manipal 576104, India; d ICMR-National Institute of Malaria Research, Field Unit, Campal, Panaji, Goa 403001, India

## Abstract

The data article reports data of the proteins expressed in female *Anopheles stephensi* salivary glands. Proteomic data were acquired using high-resolution mass spectrometers - Orbitrap-Velos and Orbitrap-Elite. Samples derived from adult female *A. stephensi* salivary glands led to the identification of 4390 proteins. Mass spectrometry data were analyzed on Proteome Discoverer (Version 2.1) platform with Sequest and Mascot search engines. The identified proteins were analyzed for their Gene Ontology annotation, interaction network and their possible roles in vector-parasite interaction. The data provided here are related to our published article “Integrating transcriptomics and proteomics data for accurate assembly and annotation of genomes” (Prasad et al., 2017) [1].

**Specifications table**

**Table:** 

Subject area	Biology
More specific subject area	Vector biology
Type of data	Excel files, figures
How data were acquired	LTQ-Orbitrap Velos ETD mass spectrometer (Thermo Scientific, Bremen, Germany)
	Proteome Discoverer 2.1 and MASCOT search engine (Matrix Science, London, UK; version 2.2)
	Protein database *Anopheles stephensi* Liston (Indian strain) (www.VectorBase.org, release February 25, 2014)
Data format	Analyzed
Experimental factors	Salivary glands were dissected from sugar fed mosquitoes and proteins extracted.
Experimental features	Proteome profiling of *Anopheles stephensi* salivary glands
Data source location	Goa and Bangalore, India
Data accessibility	Raw mass spectrometric data are available from a web application (ProteomeXchange) Consortium
	(http://proteomecentral.proteomexchange.org) via the PRIDE partner repository with the dataset identifier PXD001128.
	Analyzed data are provided along with this article as excel sheets.

**Value of the data**

• The data provide details on proteins expressed in the salivary glands of adult female *Anopheles stephensi* mosquitoes.
• The data provide an insight into the physiological processes and pathways associated in the salivary glands of *A. stephensi*.
• The data provide a platform to comprehend the possible vector-pathogen interactions occurring in the female *A. stephensi* salivary glands that may be associated with transmission of *Plasmodium*.

## 1 Data

**Fig. 1:**
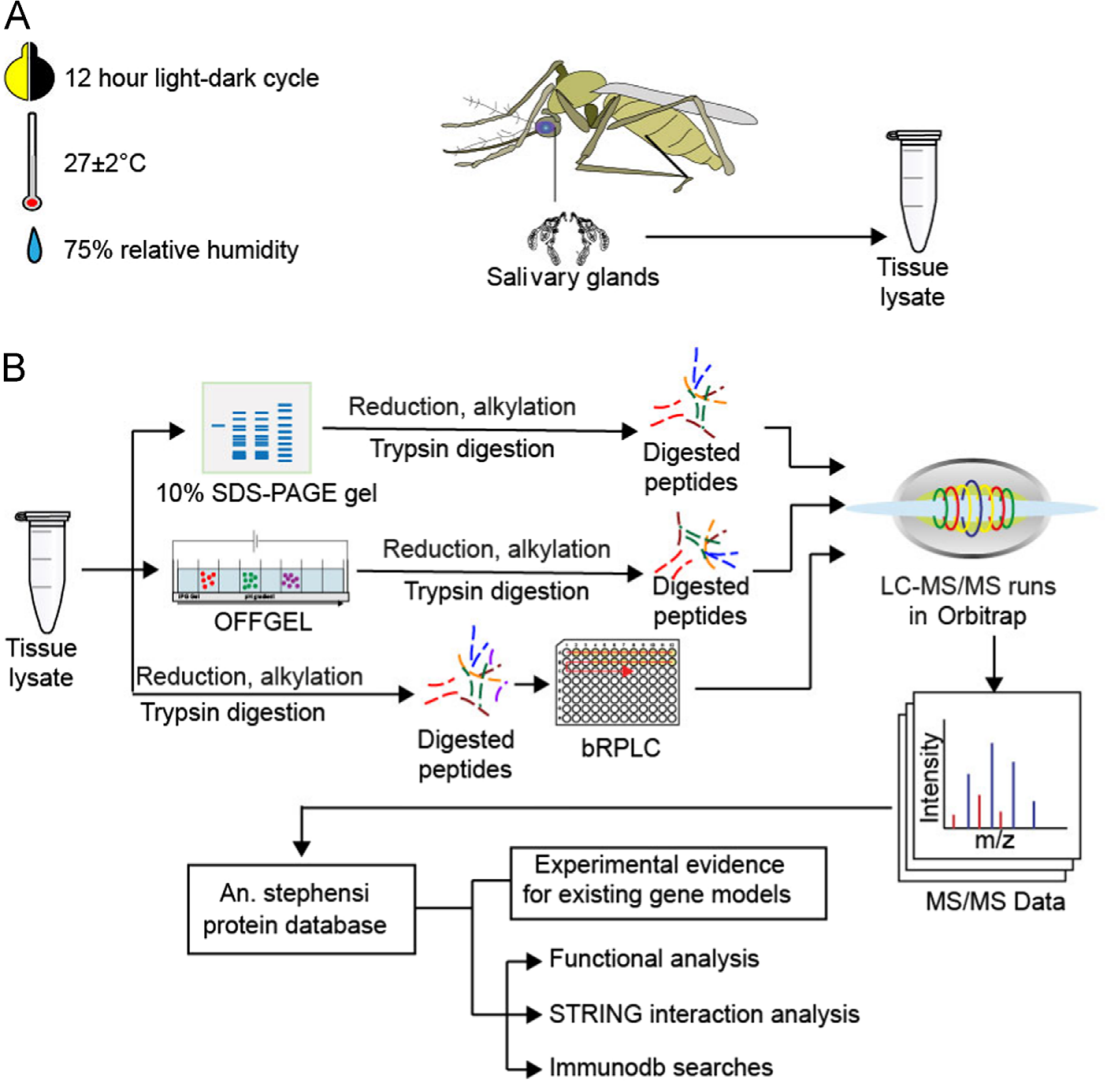
The experimental procedure carried out for the proteomic analysis of mosquito salivary glands in this study. A) Mosquito culture conditions; B) Experiment and analysis workflow.

To identify the proteins expressed inside the female *Anopheles stephensi* mosquito salivary glands, we carried out proteomic profiling of salivary glands dissected from sugar fed female *A. stephensi* (Liston strain) mosquitoes using high-resolution mass spectrometers (Fig. 1). Proteins extracted from samples were fractionated at protein level using SDS-PAGE and peptide level using basic reverse phase liquid chromatograhy (bRPLC). Fractions were then analyzed on a high-resolution mass spectrometry which resulted in the acquisition of 399,827 tandem mass spectra. These spectra were then searched against a protein database of *A. stephensi* leading to the identification of 275,522 peptide-spectrum matches (PSMs) corresponding to 38,026 unique peptides belonging to 4390 proteins. The complete list of identified proteins is provided in Supplementary Table S1.

## 2 Experimental design, materials and methods

### 2.1 Maintenance of mosquito colony

*A*. *stephensi* mosquitoes were maintained in the insectary at National Institute of Malaria Research, Field Station Goa as continuous cyclic colony at 27 ± 2 °C, 75% relative humidity and cycle of 12 h in light and 12 h in darkness. The adult female mosquitoes were fed on 10% glucose solution. Salivary glands were dissected in 0.65% normal saline under dissecting microscope and stored at −80 °C until further use.

### 2.2 Protein extraction

Homogenization of the dissected salivary glands was carried out using a probe sonicator in 200 µl of 1% SDS. The lysate was centrifuged at 14,000 rpm for 10 min at 4 °C and supernatant was collected. Protein quantification was carried out according to modified Lowry׳s method (Bio-Rad DC Protein assay) and normalized on 10% SDS-PAGE.

### 2.3 Fractionation

Proteins extracted from salivary glands were fractionated at both protein- and peptide-level as discussed previously [1], [2], [3], [4]. Fractionation at the protein-level was carried out with 300 µg of protein on 10% SDS_PAGE and 24 bands excised after Coomassie blue staining. Excised bands were reduced and alkylated with dithiothreitol (DTT) and iodoacetamide (IAA), respectively. Trypsin digestion was carried out overnight at 37 °C using sequencing grade trypsin (Promega). Digested peptides were extracted and stored at −20 °C.

Peptide-level fractionation was carried out with 500 µg of protein, which was reduced and alkylated prior to trypsin digestion at 37 °C for 16 h. Prior to bRPLC fractionation, digested peptides were cleaned using Sepak C_18_ column and lyophilized. The lyophilized peptides were reconstituted in bRPLC solvent A (10 mM triethyl ammonium bicarbonate (TEABC) in water at, pH ~9.5), loaded on XBridge C_18_, 5 µm 250 × 4.6 mm column (Waters, Milford, MA) connected to Agilent 1100 series HPLC system. The digested peptides were resolved using a gradient of 5% to 100% solvent B (10 mM TEABC in Acetonitrile, pH 9.5) in 70 min. Peptides were collected in a 96 well plate and then concatenated into 26 fractions. Fractions were dried and reconstituted in 0.1% formic acid prior to mass spectrometric analysis.

### 2.4 Mass spectrometry analysis

The fractions were reconstituted in 0.1% formic-acid prior to mass spectrometry analysis. Fractions were analyzed on LTQ-Orbitrap Velos and LTQ-Orbitrap-Elite mass spectrometers (Thermo Scientific, Bremen, Germany) interfaced with Easy-nLCII (Thermo Scienific, Bremen, Germany). Peptides were initially enriched on a reversed phase liquid chromatography (RPLC) pre-column (2 cm, 5 μ–100 Ǻ), followed by separation on an analytical column (11 cm, 3 μ–100 Ǻ) packed in-house with magic AQ C_18_ material (Michrom Bioresources, Inc, Auburn, CA). The solvent system used included 0.1% aqueous formic acid as solvent A and 95% acetonitrile,0.1% formic acid as solvent B. The peptides were loaded on to the trap column using solvent A, followed by resolution on the analytical column using a gradient of 10–35% solvent B for 75 min at a constant flow rate of 0.25 μL/min. The spray voltage and heated capillary temperature were set to 2.0 kV and 220 °C. Acquisition of data in mass spectrometer was carried out in a data dependent manner with a full scan in the range of *m/z* 350–2000. MS and MS/MS were acquired and measured using Orbitrap mass analyzer. Full MS scans were measured at a resolution 30,000 at *m/z* 400 and fifteen most intense precursors were selected for MS/MS fragmentation. Fragmentation of peptides were carried out using higher-energy collisional dissociation (HCD) method and detection range set at a mass resolution of 15,000 at *m/z* 400. The automatic gain control (AGC) for full FTMS was set to 1 million ions and for FT MS/MS was set to 0.1 million ions with maximum accumulation time of 100 ms and 200 ms.

### 2.5 Functional categorization and prediction of interaction map

Categorization of the identified proteins was performed by fetching information provided in the Panther database [5] and Cytoscape [6]. Both Panther database and Cluego [7] plugin in Cytoscape has identifiers only for *Anopheles gambiae*, we therefore, fetched the *A. gambiae* orthologs for the identified *A. stephensi* proteins using Biomart tool (version 0.7) [8] provided through VectorBase [9] (Supplementary Table S2). These *A. gambiae* identifiers were then used to fetch the Gene Ontology information. Cluepedia [10] plugin in Cytoscape was used to for the generation of the association map between genes and their biological processes.

Protein–protein interaction map of the identified proteins was generated using STRING (Search Tool for the Retrieval Interacting Genes/Proteins) online tool (version 10.5).plugin (version 1.1.0) [11].

### 2.6 Data analysis

The mass spectrometry derived data were searched against a database of 11,789 *A. stephensi* proteins obtained from VectorBase. The database search workflow consisted of SEQUEST and MASCOT search engines incorporated in the Proteome Discoverer suite, version 2.1 (Thermo Fischer Scientific, Bremen, Germany). Trypsin was used as the enzyme with a single missed cleavage allowed and a minimum peptide length of 6 amino acids. Modifications on peptides were set to static for carbamidomethylation of cysteine and variable for oxidation of methionine. Results were generated using a 1% false discovery rate (FDR) at the peptide level.

**Fig. 2:**
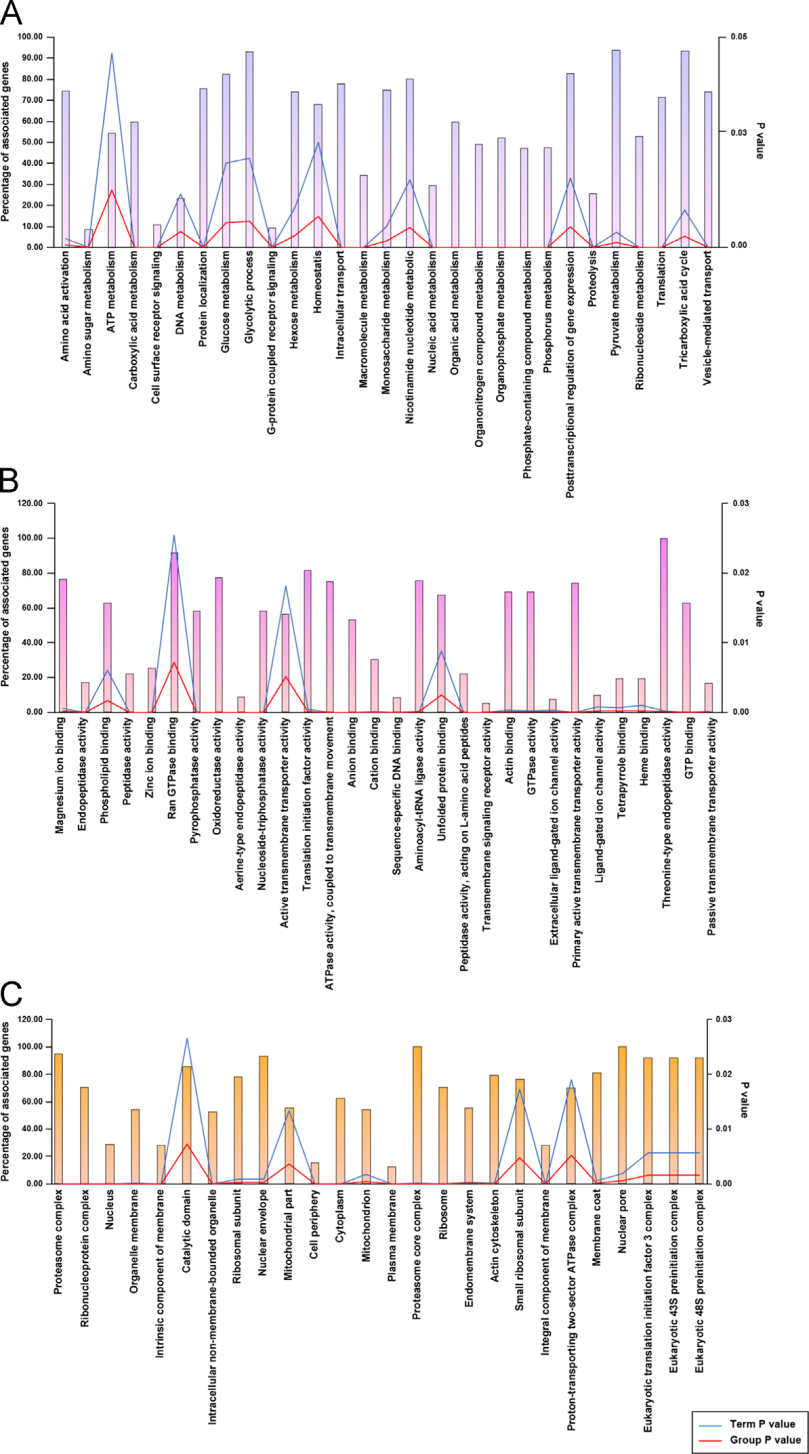
Gene ontology annotation for Proteins identified from *A. stephensi* salivary glands for A) biological processes; B) molecular functions; C) sub-cellular localization. Gene ontology terms with significant p-value were considered. Blue line represent GO term p-value and red line represents group p-value.

The proteins identified were analyzed for their role in biological processes, molecular functions and their location in cellular components. Majority of the identified proteins belonged to the protein class of nucleic acid binding proteins (17.4%), hydrolases (17.1%), transferases (9.3%) and oxidoreductases (8.5%). Functional annotations categorized the identified proteins to be associated with biological processes such as cellular processes (32.4%), metabolism (29.2%), and localization (10%). The major processes and functions of the identified salivary glands proteins have been illustrated in (Fig. 2) and a detailed Gene Ontology categorization of the identified proteins are provided in Supplementary Table S3.

**Fig. 3:**
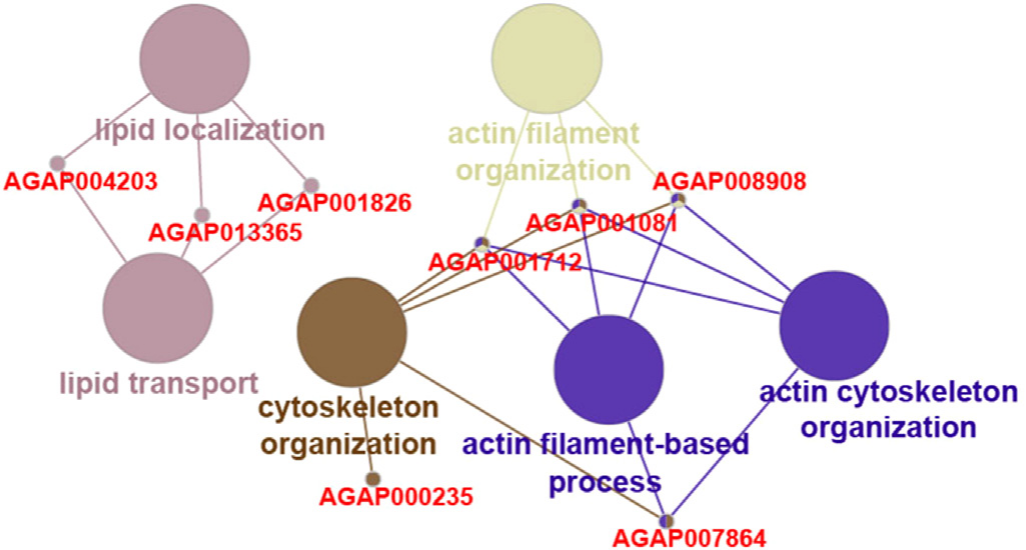
Predicted protein–protein interaction map and associated biological processes and pathways of proteins identified in the *A. stephensi* salivary glands whose *A. gambiae* orthologs were found to have vital role in vector–pathogen interactions.

**Fig. 4:**
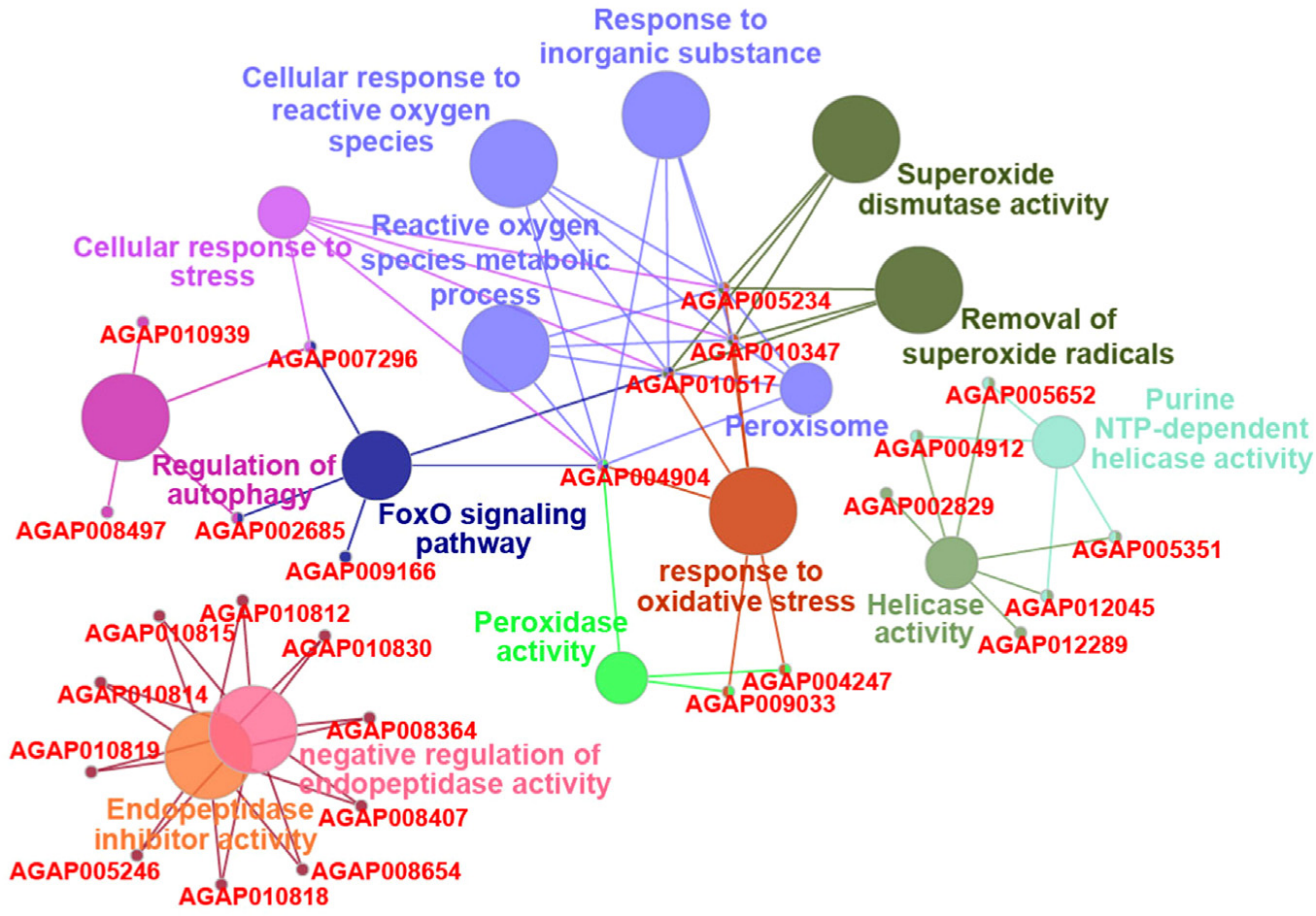
Predicted protein–protein interaction map of proteins identified in salivary glands and having a potential role in immunity (predicted by mapping to ImmunoDB database).

**Fig. 5:**
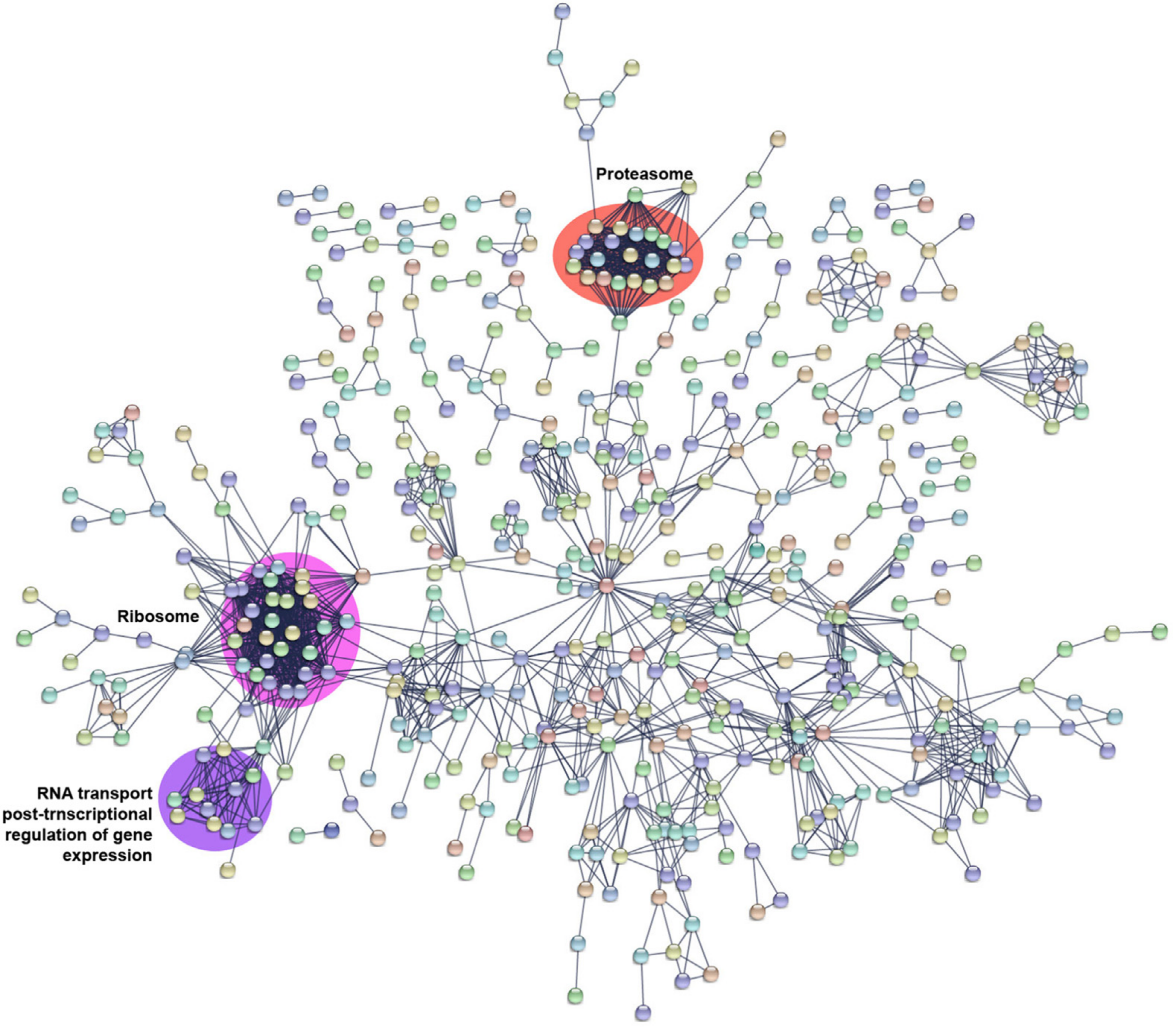
Predicted protein–protein interaction map of proteins identified in the *A. stephensi* salivary glands. The interaction map was generated using STRING online tool with high confidence parameters.

Certain genes in mosquitoes have been studied for their associated role as agonist or antagonist in vector-parasite interactions through gene knockdown experiments [12]. We observed 31 of such proteins to be expressed in the *A. stephensi* salivary glands. Among these, 30 proteins were also identified in midgut, fatbody, ovary and brain [1], [13], [14], [15]. We observed the expression of *ASTEI03572* gene to be enriched only in the *A. stephensi* salivary glands. The *A. gambiae* ortholog (*AGAP000151*) for *ASTEI03572* has been observed to show increased expression in response to blood-feeding and *Plasmodium* infection. The list of proteins identified in salivary glands that mapped to the list of proteins with experimentally proven roles in parasite development inside the mosquito is provided in Supplementary Tables S4 and S5 (Fig. 3). Development and transmission of the *Plasmodium* parasite is regulated by the immunological processes occurring inside the mosquito. Immunodb database provides information on immune-related gene families. Eighty-one proteins identified in salivary glands were found to have probable role in the generation of mosquito related immune responses. The list of proteins mapping to Immunodb along with their associated biological processes are provided in Supplementary Tables S6 and S7 (Fig. 4). To elucidate the interaction potential of the identified proteins in the salivary glands, we selected 1,006 proteins with a minimum of 10 PSMs and at least 10 unique peptides for a higher confident data. A predicted interaction map for the selected proteins was generated using Online STRING tool (Fig. 5, Supplementary Table S8).

## Appendix A Supplementary material

Supplementary Table S1: List of LC-MS/MS identified proteins in salivary glands of *A. stephensi* using high-resolution mass spectrometry.

.

Supplementary Table S2: List of proteins identified in *A. stephensi* (Indian strain) salivary glands along with their orthologs in *A. stephensi* (SDA500 strain) *A. gambiae*. Ortholog information was fetched from VectorBase.

.

Supplementary Table S3: Detailed functional analysis for the Gene Ontology terms (biological processes, molecular function and cellular component) for the identified salivary glands proteome of *A. stephensi*.

.

Supplementary Table S4: List of proteins identified in salivary glands of *A. stephensi* that were experimentally validated in *A*. *gambiae* for their association with the development of *Plasmodium* inside the mosquito vector.

.

Supplementary Table S5: List of Gene Ontology terms for proteins identified in salivary glands of *A. stephensi* that were experimentally validated in *A. gambiae* for their association with the development of *Plasmodium* inside the mosquito vector.

.

Supplementary Table S6: List of proteins in *A. stephensi* salivary glands that mapped to the data provided in Immunodb and may have potential immune related functions.

.

Supplementary Table S7: List of Gene Ontology term for proteins in *A. stephensi* salivary glands that mapped to the data provided in Immunodb and may have potential immune related functions.

.

Supplementary Table S8: List of predicted interacting proteins identified in salivary glands of *A. stephensi* using online STRING tool.
